# Sub-ppb H_2_S Sensing with Screen-Printed Porous ZnO/SnO_2_ Nanocomposite

**DOI:** 10.3390/nano14211725

**Published:** 2024-10-29

**Authors:** Mehdi Akbari-Saatlu, Masoumeh Heidari, Claes Mattsson, Renyun Zhang, Göran Thungström

**Affiliations:** Department of Engineering, Mathematics and Science Education, Mid Sweden University, Holmgatan 10, SE-85170 Sundsvall, Sweden

**Keywords:** ZnO/SnO_2_ nanocomposite, gas sensor, H_2_S, screen printing, ultrasonic spray pyrolysis

## Abstract

Hydrogen sulfide (H_2_S) is a highly toxic and corrosive gas commonly found in industrial emissions and natural gas processing, posing serious risks to human health and environmental safety even at low concentrations. The early detection of H_2_S is therefore critical for preventing accidents and ensuring compliance with safety regulations. This study presents the development of porous ZnO/SnO_2_-nanocomposite gas sensors tailored for the ultrasensitive detection of H_2_S at sub-ppb levels. Utilizing a screen-printing method, we fabricated five different sensor compositions—ranging from pure SnO_2_ to pure ZnO—and characterized their structural and morphological properties through X-ray diffraction (XRD) and scanning electron microscopy (SEM). Among these, the SnO_2_/ZnO sensor with a composition-weight ratio of 3:4 demonstrated the highest response at 325 °C, achieving a low detection limit of 0.14 ppb. The sensor was evaluated for detecting H_2_S concentrations ranging from 5 ppb to 500 ppb under dry, humid air and N_2_ conditions. The relative concentration error was carefully calculated based on analytical sensitivity, confirming the sensor’s precision in measuring gas concentrations. Our findings underscore the significant advantages of mixture nanocomposites in enhancing gas sensitivity, offering promising applications in environmental monitoring and industrial safety. This research paves the way for the advancement of highly effective gas sensors capable of operating under diverse conditions with high accuracy.

## 1. Introduction

H_2_S is a highly toxic and flammable gas, characterized by its infamous rotten-egg odor [1,2]. Even at low concentrations, H_2_S poses significant health risks, including respiratory irritation and dizziness, and, at higher concentrations, it can be fatal [3]. Additionally, H_2_S is corrosive and can cause severe damage to equipment and infrastructure, particularly in industries such as petrochemicals, wastewater treatment, and natural gas processing [4]. The ability to accurately detect and monitor H_2_S levels is therefore crucial for ensuring safety in both industrial and environmental settings. This drives the motivation to develop highly sensitive and selective sensors capable of detecting H_2_S at very low concentrations.

Metal-oxide semiconductors have long been recognized as exceptional materials for gas-sensing applications due to their high sensitivity to trace gas concentrations, stability, low cost, and potential for miniaturization and low power consumption [5,6,7]. However, while single metal-oxide sensors offer broad gas-detection capabilities, they often lack selectivity, presenting a significant challenge in distinguishing between different gases [8,9]. To address this, recent research has focused on enhancing sensor selectivity through various methods, including doping with catalytic metals [10], surface modifications [11], and the development of multicompositional sensing films [12,13].

One particularly promising approach involves the use of heterostructures composed of dissimilar metal oxides, such as ZnO and SnO_2_ [14,15]. These heterostructures leverage the formation of depletion layers at the junctions between different oxides, which can be modulated by the presence of target gases, thereby altering the sensor’s conductivity [16]. Additionally, combining two different metal oxides can increase gas adsorption sites, further improving sensor performance [17,18]. Among various combinations, ZnO/SnO_2_ heterostructures have shown remarkable improvements in sensitivity and selectivity, particularly for detecting hazardous gases like hydrogen sulfide (H_2_S) [19,20,21].

In previous studies, the authors have shown that ultrasonic spray pyrolysis (USP) can be used to deposit densely packed thin films of ZnO/SnO_2_ heterostructures for H_2_S detection [22]. These densely packed thin films achieved a response approximately 95 times better than the pure-SnO_2_ sensor for 5 ppm H_2_S at an operating temperature of 450 °C, with the lowest detection concentration of 0.5 ppm. Also, in [23], Zhu et al. developed hierarchical and highly ordered nanobowl ZnO/SnO_2_ gas sensors, which demonstrated high sensitivity and selectivity for detecting H_2_S gas at concentrations as low as 1 ppm, with long-term stability and repeatability. The hierarchical sensing materials were synthesized through a sequential process that involved hard-template processing, atomic layer deposition, and hydrothermal processing. Additionally, in [24], Guo et al. conducted a study on the hydrothermal synthesis of ZnO/SnO_2_ for H_2_S detection. Their findings showed that this type of heterostructure exhibits a better H_2_S gas response and selectivity compared to other interfering gases such as NO, SO_2_, CO, CH_4_, and C_2_H_5_OH. The most important works concerning the gas sensing of ZnO/SnO_2_ heterostructures are summarized in Table 1. In addition, recent advancements in room-temperature H_2_S sensors have gained attention. For example, Zhu et al. [25] developed a triboelectric respiration sensor (TRS) incorporating an Fe^2+^-doped polypyrrole film, which selectively reacts with H_2_S. This sensor achieved a response of 25.21% for 10 ppm H_2_S with good repeatability and a detection limit of 1 ppm, highlighting the potential of TRS technology for room-temperature H_2_S detection.

In this study, ZnO/SnO_2_ porous nanocomposites were successfully fabricated for the first time using a screen-printing method with a Mayer bar to detect sub-ppb levels of H_2_S. Five distinct sensor compositions—ranging from pure SnO_2_ to pure ZnO—were produced, and the structural, morphological, and gas-sensing properties of these sensors were thoroughly investigated. The heaters, essential for achieving the optimal operational temperature, were fabricated using USP, which enabled precise and uniform deposition on the sensor substrate. The choice of USP heaters is based on their ability to produce thick films of SnO_2_, which serve as effective and cost-effective heating elements suitable for high-temperature operations in harsh and humid environments, overcoming the limitations of conventional materials like RuO_2_ and noble metals [34,35,36]. This study shows that a 3:4 SnO_2_/ZnO ratio (in terms of sensor composition) demonstrated the highest gas-sensing performance among the investigated samples for low concentrations of H_2_S (5 ppb). To quantitatively evaluate sensor performance, the concept of analytical sensitivity was employed. Analytical sensitivity provides a robust measure of the sensor’s ability to detect small changes in gas concentration. This approach, which involves selecting the sensor signal based on the most stable and sensitive parameter (R_g_ or R_o_/R_g_), allows for the optimization of sensor performance. The results of this study highlight the potential of screen-printed porous ZnO/SnO_2_-nanocomposite thick films in developing highly efficient gas sensors with low detection limits and high sensitivity for various applications.

## 2. Experimental Details

The substrates used for fabricating ZnO/SnO_2_ porous nanocomposite sensors were high-purity (99%) alumina plates, laser cut into small dimensions of 3 mm × 3 mm × 0.5 mm. Prior to any deposition, these substrates underwent a cleaning process involving ultrasonic baths in acetone and distilled water, followed by air drying.

For microheater formation, the cleaned alumina substrates were directly placed on the hot stage of an USP system, previously reported for various applications [37,38,39]. The precursor solution, consisting of 0.5 M stannous chloride dihydrate dissolved in 99.9% (*v*/*v*) ethanol, was prepared for USP deposition. The deposition process was carried out with a spray rate of 4 mL/min at a substrate temperature of 325 °C for 30 min [40,41]. Post-deposition, the samples were annealed at 900 °C for one hour in air to stabilize the microheater characteristics, particularly for high-temperature operations.

The screen-printing method was employed to deposit the ZnO/SnO_2_ sensing layers onto the substrates [42]. A homogeneous paste was prepared by grinding a mixture of SnO_2_ and/or ZnO powder (Sigma-Aldrich, <100 nm particle size, St. Louis, MO, USA) with 1,2-propanediol (Sigma-Aldrich, 99.5+% ACS reagent) using a mortar. The resulting paste, with an oily/honey-like consistency, was coated onto the substrates using a Mayer-bar coater equipped with a 16 µm grooved metallic bar. The bar was rolled over the alumina substrates at a speed of 20 mm/s, producing a uniform layer of the sensing material. The coated sensors were then left to settle at room temperature for 1 h and subsequently dried on a hotplate at 80 °C overnight. Finally, the sensors were annealed at 500 °C for 10 min. The fabricated sensor details are listed in Table 2.

The gas-sensing properties of the fabricated sensors were evaluated in a controlled environment. The sensors were placed on a ceramic foundation equipped with electrical feedthroughs, housed within a stainless-steel chamber of 5 cm^3^ volume. The chamber, maintained in a cleanroom environment, was fitted with connectors for a gas inlet and outlet.

The gas flow was controlled using mass flow controllers (MFCs), with a continuous flow of 500 ± 5 mL/min, monitored by an independent flowmeter. Precleaned and dried compressed air was used as the carrier gas. Target gas mixtures, including 100 ppm and 1 ppm H_2_S diluted in N_2_, were introduced from standard analytical-quality gas cylinders. The relative humidity (RH) during the gas-sensing tests was carefully controlled using a bubble humidifier in combination with an MFC. By adjusting the flow rates of dry and humidified gases through the MFC, different RH levels were achieved with precision. The RH of the gas mixture was continuously monitored in real time using a commercial humidity sensor (Honeywell HIH-400-004, Honeywell, Golden Valley, MN, USA) to ensure stability and accuracy throughout the testing process. This setup allowed for the controlled evaluation of sensor performance under varying humidity conditions, which is critical for assessing sensor reliability in real-world applications.

The sensors’ operating temperature was held steady at 325 °C during measurements, with temperature monitored using a small s-type thermocouple. A constant voltage of 5 V was applied, and electrical parameters were measured using Keithley 2410 electrometers (Tektronix, Bracknell, UK). The entire experiment was controlled via LabVIEW software (version 18.0 (64-bit)), with data acquisition at 0.5 s intervals.

In gas sensing, the sensor response can be defined either by the relative change in resistance (R_o_/R_g_) or directly by the resistance in the presence of the target gas (R_g_). The choice of signal significantly affects the accuracy and reliability of the sensor readings.

Analytical sensitivity is a measure of the ability of the sensor to discriminate or detect small changes in concentration at the concentration of interest. It allows us to quantitatively compare the sensor performance with sensor responses that are different in nature and/or magnitude [43]. It is defined as the ratio of the sensor’s sensitivity (slope of the calibration curve) to the standard deviation of the sensor signal at the concentration of interest. By calculating analytical sensitivity, one can directly measure the amount of relative concentration error.

Following the approach detailed in [44], the low detection limit (LOD) was calculated by extrapolating the fitted calibration curve to its intersection with a signal level equivalent to three times the standard deviation of the noise level.

## 3. Results and Discussion

**Figure 1:**
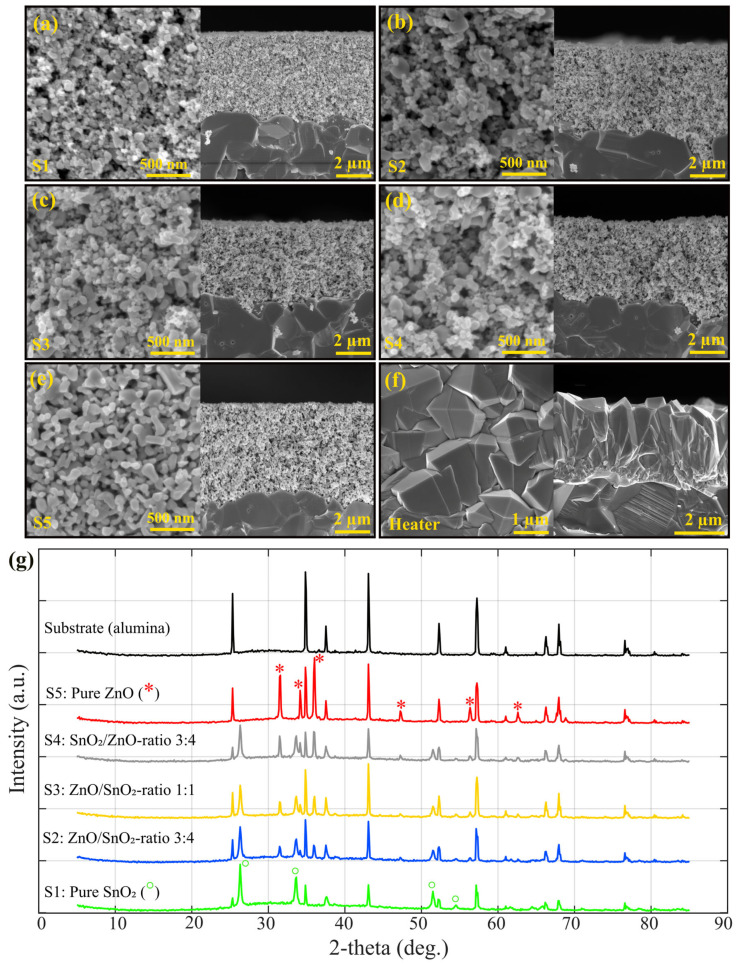
Plan view and the cross-sectional SEM micrographs of the porous thick film sensors prepared by Mayer-bar coater on alumina substrate: (**a**) S1 pure SnO_2_, (**b**) S2, (**c**) S3, (**d**) S4, (**e**) S5 pure ZnO, and (**f**) SnO_2_ densely packed thick films microheaters prepared by USP and (**g**) θ–2θ diffractograms from sensors S1 to S5 as well as alumina substrate (the peaks are represented by red * and green ° in the XRD pattern representing ZnO and SnO_2_ peaks respectively).

The SEM images of the sensors (Figure 1a–e) indicate porosity in all compositions, with a consistent film thickness of approximately 4–5 µm across the samples. The porous networks observed in the SEM micrographs are well-suited for gas diffusion. Additionally, the SEM image of the heaters (see Figure 1f) shows a uniform dense structure designed specifically by USP to provide efficient heating across the sensor surfaces.

The crystallographic phases of the fabricated ZnO/SnO_2_-nanocomposite sensors were analyzed by XRD, with distinct patterns observed for the pure-ZnO and -SnO_2_ samples as well as their composites. According to Figure 1g, for the pure-ZnO sample, the XRD pattern showed prominent diffraction peaks at 2θ values of 31.57°, 34.15°, 36.03°, 47.23°, and 56.35°, corresponding to the (100), (002), (101), (102), and (110) planes of the hexagonal wurtzite structure of ZnO (JCPDS No. 36-1451) [15,45]. These peaks confirm the successful formation of highly crystalline ZnO with no other impurity phases present (the peaks are represented by red * in the XRD pattern of ZnO). The well-defined peaks indicate a high degree of crystallinity in the ZnO layer. In contrast, the pure-SnO_2_ sample exhibited diffraction peaks at 2θ values of 26.31°, 33.65°, 51.5°, and 54.57° corresponding to the (110), (101), (211), and (220) planes, respectively. These reflections are characteristic of the tetragonal rutile phase of SnO_2_ (JCPDS No. 41-1445), confirming the high crystallinity of the SnO_2_ component (the peaks are represented by green ° in the XRD pattern of SnO_2_) [46,47].

In the composite sensors, the XRD patterns reflected a combination of the distinct ZnO and SnO_2_ peaks, depending on the ZnO and SnO_2_ ratio. For instance, in the S4 (SnO_2_/ZnO, ratio 3:4) sample, peaks from both the hexagonal ZnO and the tetragonal SnO_2_ phases were detected, indicating the coexistence of both materials without significant phase interaction or the formation of secondary compounds. The preservation of these individual crystalline phases suggests that the heterostructures (in the form of mixture nanocomposites) were formed via a simple physical mixing of the ZnO and SnO_2_ powders without a solid-state reaction at the temperatures used in this study. The S4 sample, which exhibited both the ZnO and SnO_2_ diffraction peaks, showed the highest H_2_S gas response, suggesting that the presence of both phases is essential for enhancing gas-sensing performance.

**Figure 2:**
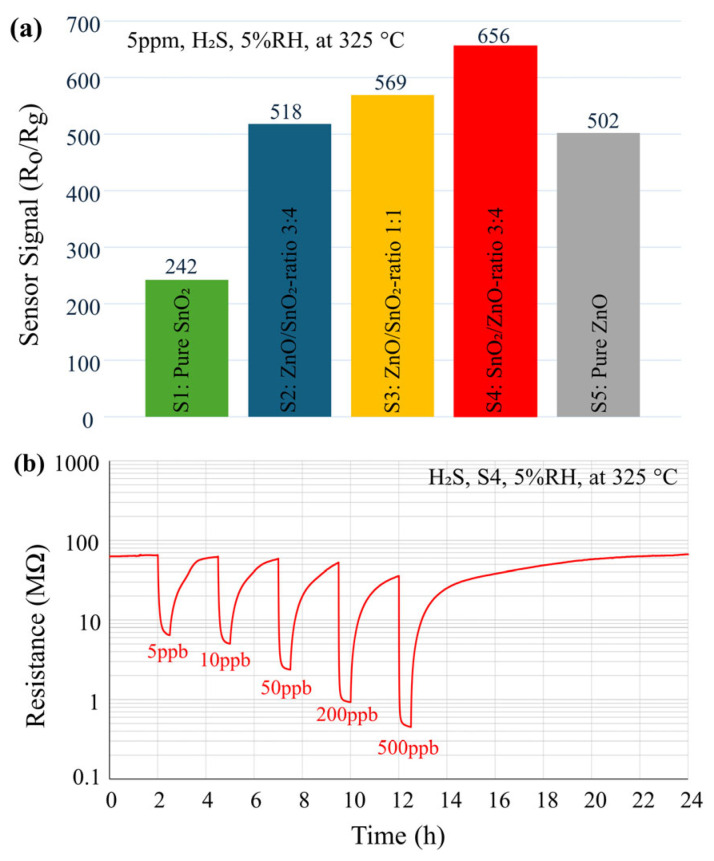
(**a**) Sensor signal (R_o_/R_g_) of all sensors (S1 to S5) towards 5 ppm of H_2_S. (**b**) Dynamic response (R_g_) of the sensor S4 to different concentrations of H_2_S (5, 10, 50, 200, 500 ppb, exposure time is 30 min) in dry condition (5%RH).

In order to determine the optimal operating temperature, sensor S3 was exposed to 5 ppm of H_2_S for 5 min at varying temperatures, ranging from 200 °C to 450 °C (at 5% RH). Due to the baseline fluctuations, the same procedure was applied at each temperature: a 20-minute waiting time before introducing the target gas, followed by a 5-minute exposure. The highest sensor response was observed at 325 °C (See Figure S2). This temperature is lower than our previous findings for ZnO/SnO_2_ heterostructures prepared by USP [22]. At lower operating temperatures, the gas molecules do not have enough energy to overcome the activation energy required for reacting with the oxygen species on the surface of ZnO/SnO_2_, resulting in a lower response. As the temperature increases, the surface-adsorbed oxygen species are more easily converted, and the reaction activity rises, leading to a higher response. However, when the temperature goes beyond the optimal level, H_2_S gas adsorption is too difficult to be adequately compensated for by the increased surface reactivity, which causes a low utilization rate of the sensing material [48]. Additionally, although higher temperatures result in shorter response and recovery times, the overall response decreases, and more energy is needed due to the higher temperature. The lower optimal operating temperature of 325 °C compared to [22] can be explained by the enhanced gas diffusion and interaction within the nano-porous structure of the ZnO/SnO_2_ film. The optimal operation temperature was determined by the reaction between the oxygen adsorbates and the target gas [49]. By using porous ZnO/SnO_2_ structures instead of dense ones, there are more adsorption sites available for oxygen molecules due to the increased surface area and open pathways within the porous matrix. This larger surface area enhances the interaction between the sensor material and the target gas, allowing for more efficient adsorption and reactions at lower temperatures. As a result, the optimal operating temperature is reduced because the increased number of active sites in the porous structure facilitates the reaction between the adsorbed oxygen species and the target gas more effectively. As illustrated in Figure 2a, all sensors were subsequently tested at this optimal operating temperature on 5 ppm of H_2_S for 5 min under dry conditions (5% RH). This time, to ensure a more stable baseline, a longer waiting time of 2 h was applied before introducing the target gas. The results indicate that sensor S4 exhibited the highest response among the investigated samples and was therefore selected for further investigation. Interestingly, in all compositions containing both ZnO and SnO_2_ (S2, S3, S4), the response was higher than that of the pure-SnO_2_ (S1) and pure-ZnO (S5) samples, highlighting the critical role of nanocomposites in enhancing the sensor’s response to the target gas.

The dynamic response (R_g_) of the sensor (S4) to H_2_S gas is illustrated in Figure 2b. The sensor is exposed to varying concentrations of H_2_S (5 ppb, 10 ppb, 50 ppb, 200 ppb, and 500 ppb) at 325 °C for 30 min in 5% RH, followed by a 2-hour recovery period. The sensor (S4) exhibited changes in resistance (R_o_/R_g_) of approximately 10, 12, 28, 60, and 82 times upon exposure to 5 ppb, 10 ppb, 50 ppb, 200 ppb, and 500 ppb of H_2_S, respectively. These values (of detection levels and concentrations) are significantly lower than those reported in previous studies [19,22].

**Figure 3:**
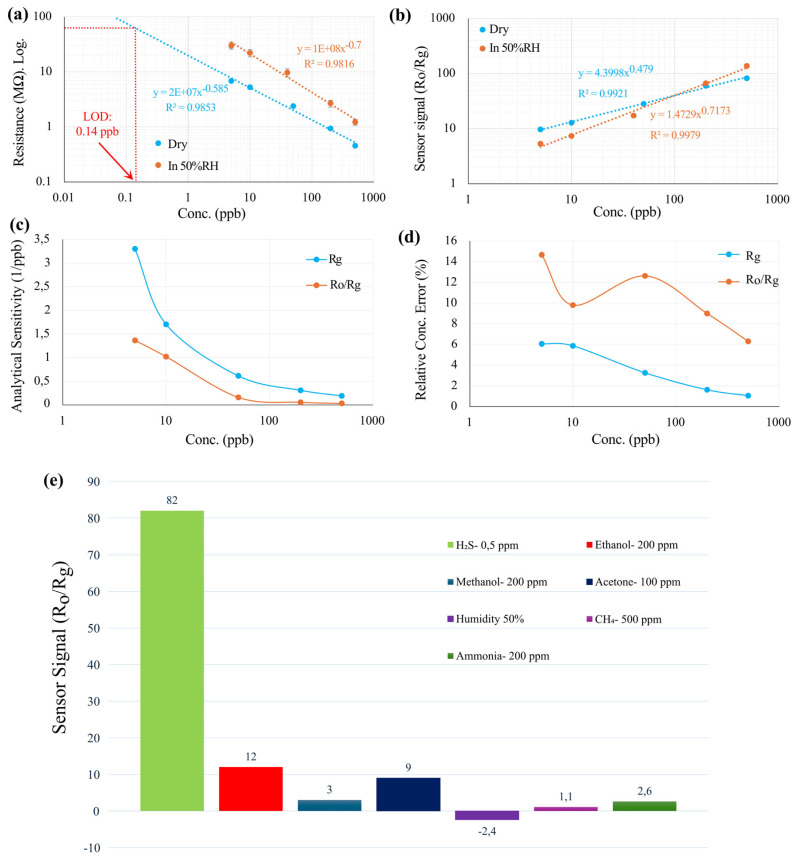
The fitting curve of the sensor S4 in response to H_2_S (5 ppb to 500 ppb) under dry (5%RH) and humid condition (50%RH) for the (**a**) resistance of the sensor (R_g_) and (**b**) relative changes of the resistance (R_o_/R_g_). (**c**) Calculated analytical sensitivity in dry condition and (**d**) corresponding relative concentration error. (**e**) Selectivity of the S4 towards some interfering gases at 325 °C (5%RH).

The six measured points and corresponding fitting curves (sensor signal vs. concentration) for R_g_ and R_o_/R_g_ are presented in Figure 3a,b. To further investigate the sensor signal, the analytical sensitivity (sensitivity over the standard division of sensor signal) is calculated and presented in Figure 3c, indicating higher values for R_g_ compared to R_o_/R_g_ at all concentrations. In this figure, both curves related to analytical sensitivity decrease gradually by increasing the concentration.

An analysis of the sensor’s performance reveals that using R_o_/R_g_ leads to higher error bars compared to R_g_. This is primarily due to variations in the baseline resistance (R_o_), which can introduce significant inaccuracies, particularly at low concentrations. To be more precise, as demonstrated in Figure 3d, the relative concentration error with R_o_/R_g_ reached up to 14% at 5 ppb, highlighting substantial measurement uncertainties. Conversely, when R_g_ is used as the sensor signal, the influence of baseline fluctuations is minimized, resulting in a more stable and accurate detection of the target gas. This approach yields lower relative concentration errors, generally less than 6% across all tested concentrations (5 ppb to 500 ppb), and reaching around 1% relative concentration error at 500ppb, as illustrated in Figure 3d. The improved precision with R_g_ underscores its advantage over R_o_/R_g_ in reducing measurement errors and enhancing sensor performance. Thus, selecting R_g_ as the sensor signal is crucial for achieving a more accurate and reliable response. By mitigating baseline-related inaccuracies, R_g_ provides a more effective measurement of target gases, ensuring higher sensitivity and precision in gas-detection applications.

Figure 3e illustrates the sensor’s performance, highlighting its enhanced selectivity for H_2_S over common interfering gases like ethanol, CH_4_, methanol, acetone, ammonia, and humidity. The selectivity of the ZnO/SnO_2_ sensor for H_2_S detection can be attributed to several key factors. One significant reason is the relatively small molecular size of H_2_S compared to other gases. This smaller size results in a greater adsorption capacity on the available surface area of the sensor, enhancing its sensitivity to H_2_S [23]. Moreover, previous research conducted by Fu et al. [19] demonstrated that ZnO reacts with H_2_S, leading to the formation of ZnS. This transformation is critical because ZnS possesses higher conductivity than ZnO. As a result, the sensor exhibits a larger response when detecting H_2_S due to this increased conductivity. This finding confirms that the ZnO/SnO_2_ is particularly effective for selective H_2_S detection, underscoring its potential applications in environmental monitoring and safety.

To examine the effect of humidity, the same measurements were performed at 50% RH, and the results, along with the fitted curves, are shown in Figure 3a,b. The data indicate that higher humidity levels cause changes in the sensor’s resistance. However, when using the sensor’s resistance (R_g_) as the sensor signal, the slope of the calibration curve, or sensitivity, in humid conditions remains almost the same as in dry conditions. On the other hand, when using R_o_/R_g_ as the sensor signal, the sensitivity changes significantly under humid conditions compared to the dry state (5% RH). In humid environments, H_2_S molecules must compete with water molecules for adsorption sites on the pre-adsorbed oxygen species. This suggests that in humid conditions, fewer sites are available for H_2_S molecules to adsorb and contribute to the sensor’s conductivity.

The LOD for S4 (5%RH, at 325 °C, for H_2_S) was determined to be 0.14 ppb. In addition, detailed information regarding the response and recovery times of sensor S4 is provided in Table S1 in the Supplementary Information. These calculations demonstrate the sensitivity of the sensor and its capability to detect low concentrations of these gases with a high degree of confidence.

### Sensing Mechanism

Oxygen plays an essential role in redox reactions on the surface of metal oxides [50]. When the sensor is exposed to air, oxygen molecules are absorbed on its surface, leading to the formation of reactive oxygen species such as O_2_^−^, O^−^, and O^2−^ [51,52]. These species are formed as electrons and move from the metal-oxide surface to the adsorbed oxygen, causing the surface to oxidize and resulting in upward-band bending in the energy diagram. At 325 °C, O^−^ is the main species on the surface. When the sensor comes into contact with a reducing gas like H_2_S, these oxygen species react with the gas, releasing electrons into the sensing material. The reaction can be written as follows:

H_2_S + 3o^−^_(ads)_ → SO_2_ + H_2_O +3e^−^ at 325 °C

**Figure 4:**
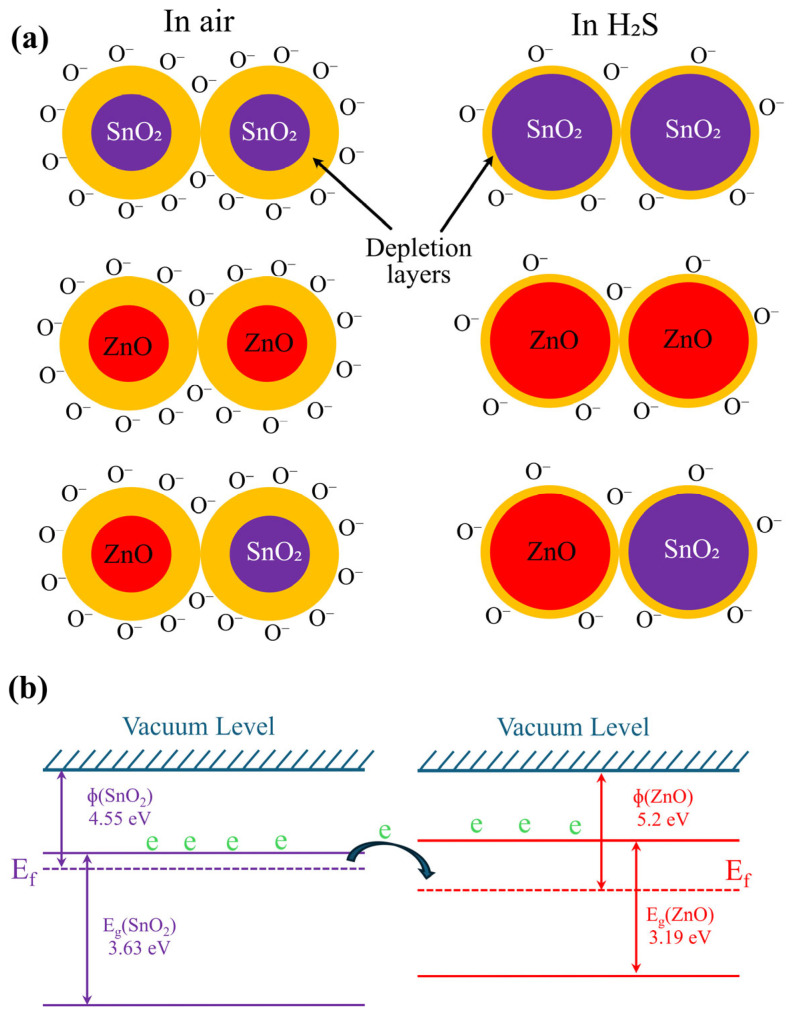
(**a**) Gas-sensing mechanism in air and H_2_S, (**b**) energy band diagram of ZnO and SnO_2_ at equilibrium state before contact and after contact fermi levels will be equal.

This reaction increases the number of free electrons on the surface, thereby reducing the sensor’s resistance. In the presence of air, a depletion layer forms on the ZnO/SnO_2_ grains, primarily controlled by negatively charged oxygen species. Electrons must overcome the barrier between the dissimilar and similar grains (ZnO grains, SnO_2_ grains, and ZnO/SnO_2_ grains—see Figure 4a. Also, a related energy-band diagram is presented in Figure 4b) to contribute to electrical conduction. The baseline resistance of the nanocomposites (ZnO/SnO_2_) is significantly higher than that of the pure ZnO or SnO_2_, indicating the existence of an energy barrier between the dissimilar metal-oxide grains [17].

When the sensor is exposed to H_2_S, the adsorbed oxygen species on the surface of ZnO/SnO_2_ grains react with the gas molecules, releasing free electrons. In addition, thanks to the porous structures prepared by screen printing, H_2_S can penetrate into the sensing layer and interact with the whole sensing layer. This interaction reduces the thickness of the depletion layer (see Figure 4a) and lowers the barrier at the nanocomposite interface, allowing electrons to flow more easily across the grain boundaries. As the barrier decreases, the resistance of the sensor drops, leading to an increase in current flow. This is especially evident in ZnO/SnO_2_ nanocomposites, where the combination of both materials enhances the sensitivity and response to H_2_S due to the formation of additional higher barriers in between dissimilar grains compared to the barrier formed between similar grains. To be more precise, when ZnO and SnO_2_ are combined, a heterojunction is established, which modifies the distribution of surface charges and induces band bending at the surface. This effect arises from the inherent differences in work functions and band gaps of ZnO and SnO_2_, which leads to electron transfer between the two components upon contact.

To better understand the electronic interactions within the interface, it is essential to consider the band gaps and work functions of both materials. According to the literature, ZnO has a band gap of 3.19 eV and a work function of 5.2 eV. In contrast, SnO_2_ has a band gap of 3.63 eV and a work function of 4.55 eV [17]. This configuration results in the ZnO/SnO_2_ nanocomposite being classified as a type of n–n heterojunction. When these two materials are in contact, the energy bands become bent due to the differences in their electronic properties.

The built-in potential at the interface between ZnO and SnO_2_ is determined by the difference between their work functions, which is approximately 0.44 eV. This built-in potential plays a crucial role in facilitating charge-carrier movement and enhancing the overall sensitivity of the gas sensor. As H_2_S molecules interact with the nanocomposites, their adsorption and desorption processes alter the surface electron states and free carrier density. This results in modifications to the band bending and built-in potential, allowing for improved detection capabilities.

For ZnO/SnO_2_ porous structures constructed using screen printing, in the presence of N_2_, the number of free charge carriers involved in conduction is equal to the number of free charge carriers in the bulk material. As a result, considering this, there is no initial band bending, and the sensor’s resistance in N_2_ sets the threshold between conduction mechanisms controlled by the depletion layer and the accumulation layer [53,54]. For the S4 sensor, this boundary is measured at 766 kΩ (for the sensor’s resistance in N_2_, see Figure S1 in Supplementary Information).

In dry air, as the concentration of the target gas increases, the sensor’s resistance decreases. For H_2_S concentrations higher than 260 ppb (according to the calibration curve), the conduction mechanism shifts from being controlled by the depletion layer to the accumulation layer. However, in humid conditions (50% RH), across all investigated H_2_S concentrations (5 ppb to 500 ppb), the conduction remains controlled by the depletion layer, with no transition to the accumulation layer observed. However, according to the calibration curve (in 50% RH), for concentrations higher than 1 ppm, the conduction mechanism will shift to the region controlled by the accumulation layer.

## 4. Conclusions

This research successfully developed ZnO/SnO_2_ porous nanocomposite gas sensors for the detection of H_2_S, with sensor S4 (SnO_2_/ZnO ratio 3:4) showing the best performance among the tested compositions. The sensor demonstrated high sensitivity at 325 °C and a LOD of 0.14 ppb, with the ability to detect H_2_S concentrations as low as 5 ppb. The relative concentration error, calculated based on analytical sensitivity, confirmed the precision of the sensor’s response by choosing the right sensor signal. Compared to pure-SnO_2_ and -ZnO sensors, the nanocomposite-based sensors exhibited enhanced gas-sensing performance. The sensor’s stable performance under both dry and humid conditions makes it promising for real-world applications. These findings provide a solid basis for the development of advanced gas sensors with high accuracy and low detection limits.

## Figures and Tables

**Table 1:** Comparative results of ZnO/SnO_2_ sensors for gas sensing.

Material	Concentration (ppb)	Response (R_a_/R_g_)	T (°C)	Target Gas	Ref.
ZnO/SnO_2_	5	9.7	325	H_2_S	This work
SnO_2_/ZnO	500	11.5	100	H_2_S	[19]
ZnO/SnO_2_	500	30	450	H_2_S	[22]
SnO_2_ promoted with ZnO	500	4.5	350	H_2_S	[20]
ZnO/SnO_2_ heterogeneous nanospheres	500	3.94	300	H_2_S	[24]
SnO_2_ promoted with ZnO	500	0.71	350	H_2_S	[21]
Au-doped ZnO/SnO_2_ nanofibers	1000	73.3	350	H_2_S	[26]
ZnO/SnO_2_ heterostructure	1000	317	350	H_2_S	[27]
SnO_2_ nanobowls branched ZnO NWs	1000	6.24	250	H_2_S	[23]
ZnO/SnO_2_ nanowires	10,000	319.6	225	H_2_S	[28]
CuO functionalized SnO_2_-ZnO core-shell NWs	10,000	1.69	RT	H_2_S	[29]
SnO_2_-ZnO core-shell NWs	25,000	3.08	400	Ethanol	[30]
SnO_2_/ZnO hierarchical nanostructures	25,000	3	400	Ethanol	[31]
ZnO/SnO_2_ nanofibers	50,000	63.3	250	H_2_S	[32]
SnO_2_-ZnO core-shell NWs	200,000	280	400	Ethanol	[33]

**Table 2:** Five distinct sensor compositions, ranging from pure SnO_2_ to pure ZnO.

Sensors	Composition Powder Weight Ratio	Explanation
S1	Pure SnO_2_	Only SnO_2_ powder
S2	ZnO/SnO_2_ 3:4	The weight of ZnO powder was three-quarters (3/4) of the weight of SnO_2_ powder
S3	ZnO/SnO_2_ 1:1	The weight of SnO_2_ powder was the same as the weight of ZnO powder
S4	SnO_2_/ZnO 3:4	The weight of SnO_2_ powder was three-quarters (3/4) of the weight of ZnO powder
S5	Pure ZnO	Only ZnO powder

## Data Availability

Data are contained within the article and Supplementary Materials.

## Supplementary Materials

The following supporting information can be downloaded at: https://www.mdpi.com/article/10.3390/nano14211725/s1, Figure S1: Dynamic response (R_g_) of the sensor S4 to different concentrations of H_2_S under 50%RH and in N_2_ conditions.; Table S1: Response and recovery time for the sensor S4 in different concentrations.; Figure S2: Response of the sensor S3 towards 5 ppm H_2_S at different operating temperature.
