# Supplementary material for: Sub-ppb H2S Sensing with Screen-Printed Porous ZnO/SnO2 Nanocomposite
Source: Nanomaterials (Basel). 2024 Oct 29;14(21):1725. doi: 10.3390/nano14211725 (PMC11547621; doi:10.3390/nano14211725)
Supplement: Supplementary file 1 [file nanomaterials-14-01725-s001.zip › nanomaterials-3262150-supplementary.pdf]

Supplementary

Sub-ppb H_2S Sensing with Screen-Printed Porous ZnO/SnO_2 Nanocomposite

Mehdi Akbari-Saatlu *, Masoumeh Heidari, Claes Mattsson, Renyun Zhang and Göran Thungström

Department of Engineering, Mathematics and Science Education, Mid Sweden University, Holmgatan 10, SE-85170 Sundsvall, Sweden
* Correspondence: mehdi.akbarisaatlu@miun.se, mehdiakbari125@gmail.com

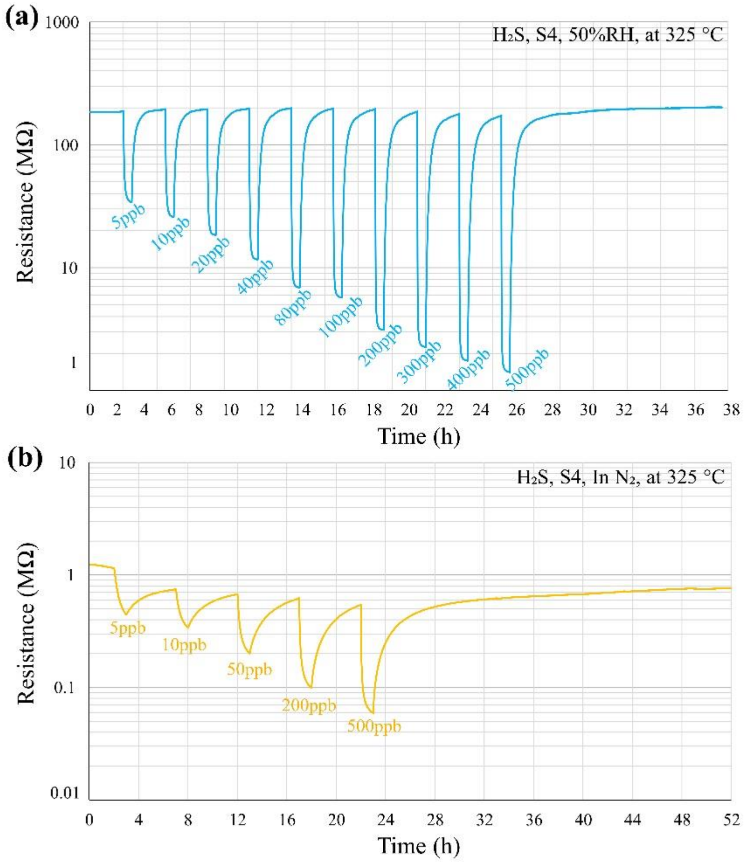

Figure S1. Dynamic response (R_g) of the sensor S4 to different concentrations of H_2S under 50%RH and in N_2 conditions.

Table S1. Response and recovery time for the sensor S4 in different concentrations.

Concentration (ppb)	Response time Rg ($T_{90\%}$)	Recovery time Rg ($T_{10\%}$)	Recovery time Rg ($T_{50\%}$)
5	321 s	5176 s	2566 s
10	217 s	7216 s	2884 s
50	85 s	>2h	3958 s
200	25 s	>2h	6058 s
500	17 s	>2h	8835 s

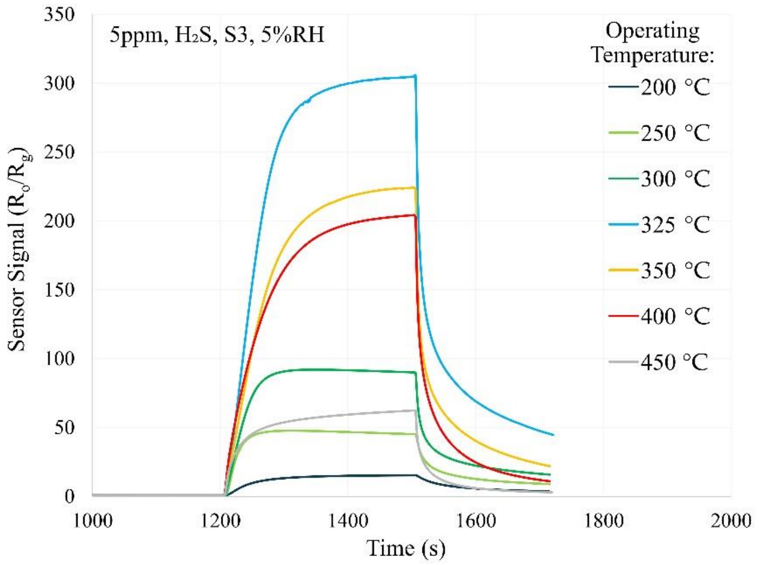

Figure S2. Response of the sensor S3 towards 5 ppm H_2S at different operating temperature.
